# Sirtuins Expression and Their Role in Retinal Diseases

**DOI:** 10.1155/2017/3187594

**Published:** 2017-01-19

**Authors:** Sankarathi Balaiya, Khaled K. Abu-Amero, Altaf A. Kondkar, Kakarla V. Chalam

**Affiliations:** ^1^Department of Ophthalmology, University of Florida College of Medicine, 580 W. 8th Street, Tower-2, Jacksonville, FL 32209, USA; ^2^The Vanderbilt Eye Institute, Vanderbilt University Medical Center, Nashville, TN, USA; ^3^Glaucoma Research Chair, Department of Ophthalmology, College of Medicine, King Saud University, Riyadh 11424, Saudi Arabia

## Abstract

Sirtuins have received considerable attention since the discovery that silent information regulator 2 (Sir2) extends the lifespan of yeast. Sir2, a nicotinamide adenine dinucleotide- (NAD-) dependent histone deacetylase, serves as both a transcriptional effector and energy sensor. Oxidative stress and apoptosis are implicated in the pathogenesis of neurodegenerative eye diseases. Sirtuins confer protection against oxidative stress and retinal degeneration. In mammals, the sirtuin (SIRT) family consists of seven proteins (SIRT1–SIRT7). These vary in tissue specificity, subcellular localization, and enzymatic activity and targets. In this review, we present the current knowledge of the sirtuin family and discuss their structure, cellular location, and biological function with a primary focus on their role in different neuroophthalmic diseases including glaucoma, optic neuritis, and age-related macular degeneration. The potential role of certain therapeutic targets is also described.

## 1. Introduction

Neurodegeneration processes are implicated in several eye diseases. These include glaucoma, age-related macular degeneration (AMD), and inherited retinal disorders [1–3]. Recently transcription factors like sirtuins were found to be involved in neurodegeneration. Identification of these cell-based markers and therapeutic modalities of neuroprotection are active areas of research in this field.

Sirtuins (silent information regulator, Sir2) were first identified to prolong lifespan in yeast* (Saccharomyces cerevisiae)*. They are originally categorized as class III histone deacetylase (HDAC) and belong to a conserved family of nicotinamide adenine dinucleotide- (NAD-) dependent protein deacylases [4]. Sirtuins deacetylate both histones and nonhistone proteins. These include transcription factors metabolic enzymes and proteins that have key roles in various cellular processes [5]. In mammals, seven human Sir2 homologues (sirtuins) designated as SIRT1 to SIRT7 have been identified to date. These are associated with calorie restriction, aging, metabolism, cancer, transcriptional silencing, chromosomal stability, stress response, cell differentiation, inflammation, apoptosis, DNA repair, and prevention of age-related ocular diseases. Sirtuins are reported to have key roles in cellular senescence, cell differentiation, and inflammation [6–11].

The aim of this review is to summarize and discuss the cellular location, biological function, and neuroprotective effect of sirtuins as a promising target for the future treatment of related neurodegenerative diseases of the eye.

## 2. Structure and Biological Function of Sirtuin

High resolution crystal structures of sirtuin family members have provided insight into their substrate, cofactor binding partners, and catalytic mechanisms [12, 13]. All the seven members of the SIRT family share a conserved catalytic core. The central catalytic core is comprised of 245 residues flanked by N- and C-terminal extensions. The core is made up of a large domain consisting of a Rossmann fold. This is typical for NAD-dependent proteins and a small Zn^2+^ ribbon motif containing the consensus sequence Cys-X_2–4_-Cys-X_15–40_-Cys-X_2–4_-Cys and a *α*-helical region. The two domains are separated by a cleft at the interface where the peptide substrate binds. SIRT1 is the most studied human isoform. It is the largest with extended N- and C-terminals and is very flexible and unstructured, which allows it to offer more sites of activity modulation (such as posttranslational modifications, interaction with proteins, and ligands). Unlike other HDACs, where zinc is part of the catalytic mechanism [14], the zinc ion is located in the small domain, far away from the NAD^+^ binding domain, excluding the possibility of its participation in the catalysis.

Since sirtuins are protein deacylases the majority of them function as deacetylases (SIRT1, SIRT2, SIRT3, SIRT5, SIRT6, and SIRT7). Their enzymatic activity results in the removal of an acetyl group from* N*-*ε*-lysine residues and generates* O*-acetyl-ADP-ribose and nicotinamide. In addition SIRT4, SIRT6, and SIRT7 exhibit monoadenosine diphosphate- (ADP-) ribosyltransferase activity. SIRT1, SIRT6, and SIRT7 are predominantly localized in the nucleus; SIRT3, SIRT4, and SIRT5 reside within the mitochondria; and SIRT2 is limited to the cytoplasm. In response to oxidative stress, shuffling of nuclear-to-cytoplasmic localization of SIRT1 has also been reported [15, 16]. Some of the major functions were shown in Table 1.

SIRT1 has been extensively studied due to its deacetylation of transcription factors and apoptotic modulators (including forkhead box O subclass (FOXO), peroxisome proliferator-activated receptor-*γ* coactivator 1*α* (PGC-1 *α*), nuclear factor kappa-B (NF-*κ*B), Ku70, and p53) [17]. It is associated with inflammation, apoptosis, genome stability, metabolic regulation, senescence, cell differentiation, and oncogenic transformation [18]. SIRT2 regulates mitotic checkpoints, oligodendrocyte, and adipocyte differentiation [15, 19]. It plays a vital role in glucose homeostasis during oxidative stress by deacetylating/activating glucose 6 phosphate dehydrogenase in pentose phosphate pathway [16]. SIRT2 is involved in the regulation of tumor necrosis factor-alpha (TNF-*α*) induced necroptosis [20]. In contrast, another study revealed that genetic and pharmacological inhibition of SIRT2 did not inhibit TNF-*α* induced necroptosis [21] suggesting that it may have a minor role.

SIRT3 has been shown to be associated with the human bladder [22] and oral squamous cell carcinoma [23]. It is a stress-responsive deacetylase, whose increased expression protects from obesity induced metabolic deregulation, cancer, and oxidative stress-mediated cell death [15, 24]. It can function either as a tumor promoter or as a tumor suppressor depending on the cell- and tumor-type and the presence of different stress or cell death stimuli [24]. SIRT3 acts as a tumor suppressor, at least in part via its ability to suppress reactive oxygen species (ROS) and regulate hypoxia inducible factor-1-alpha (HIF-1*α*) [25]. SIRT4 regulates glutamine catabolism and has lipoamidase activity that is induced by high levels of glutamine and negatively regulates pyruvate dehydrogenase complex [26]. Genetic knockdown of SIRT4 has been shown to increase SIRT1 and SIRT3 and enhance the expression of genes associated with fatty acid oxidation and mitochondrial oxidative capacity [15, 27]. SIRT5 regulates lysine succinylation, malonylation, glutarylation, enzymes involved in ketone production, and fatty acid oxidation [15, 28]. It regulates urea cycle through carbamoyl phosphate synthetase 1 via desuccinylase activity instead of deacetylase activity [29, 30]. A recent study revealed that through demalonylation of glycolytic enzymes SIRT5 positively regulates glycolysis and provides a link to cancer metabolism [29].

SIRT6, a chromatin-associated nuclear protein, promotes resistance to DNA damage and suppresses genomic instability in mouse cells, in association with a role in base excision repair [31]. Oxidative stress reduces SIRT6 levels and causes endothelial cell senescence [15, 32]. SIRT6 knockout mice display premature aging symptoms, including excessive loss of subcutaneous fat and a significant reduction in bone density, and die within 4 weeks of birth [31]. However, overexpression of SIRT6 expands lifespan in male mice by regulating insulin-like growth factor 1 (IGF-1) [32].

SIRT7 has a protective effect by deacetylating the transcription factor GA binding protein subunit beta 1 (GABP*β*1). SIRT7 deficient mice develop fatty liver and hearing loss and die prematurely from cardiomyopathy [33].

## 3. Sirtuin Expression in the Eye

Except SIRT5, all sirtuins are expressed in human retina [34, 35]. Retina is a photoreceptive tissue, whose energy consumption changes depending on light exposure. Retinal cells expend more energy in the dark due to their higher oxygen consumption and lactate production [36–38]. Hence, the retinal expression of sirtuins has been found to be variable, highlighting the regulatory mechanism(s) of sirtuins in the retina. All sirtuins showed significant daily variation under light-dark condition in retina. The mRNA levels of sirtuins except SIRT6 were elevated in dark phase. However, this photosensitive effect is absent in the brain and liver, suggesting that sirtuins may be regulated in a tissue- or organ-specific manner [38].

Jaliffa and colleagues have shown that SIRT1 is expressed in mouse cornea, lens, iris, ciliary body, inner nuclear layer, outer nuclear layer, and retinal ganglion cell layer [39]. SIRT1 deficient mice have been reported to be smaller than normal at birth and usually die during the early postnatal period. They fail to open one or both eyes [40]. In addition, multiple retinal cell layers are significantly thinner than normal mouse eyes, whereas inner and outer nuclear layers are disorganized in these mice. The difficulty in detecting inner and outer photoreceptor cell segments implicates the role of SIRT1 in ocular morphogenesis [11, 41]. In addition, SIRT1 conditional KO mice exhibit p53 hyperacetylation and reduced number of retinal neuronal cells during development [41]. To date, only a few studies reported the role of SIRT1 with the development of cataract [42, 43], retinal degeneration [44, 45], optic neuritis [46], and uveitis [47]. SIRT2 is expressed in human retinoblastoma and other nonaffected normal ocular areas such as nonpigmented ciliary body epithelium, outer and inner plexiform layer, nerve fiber layer, inner and outer nuclear layer, and retinal pigment epithelium [48]. It is also expressed in inflammatory cells at limbus and iris stroma of retinoblastoma cases [49].

SIRT3 is highly expressed in lacrimal gland and neural retina of mice mainly in retinal ganglion and photoreceptor cells [49], but 10-week-old SIRT3 knockout (KO) mice did not show any difference in retinal thickness or electroretinogram. But there is lack of evidence in neural protein expression such as rhodopsin, glial fibrillary acidic protein, and synaptophysin in these SIRT3 KO mice [50]. In humans, the inner nuclear layer (INL) showed weak expression of SIRT3 throughout the retina.

In the human retina, retinal pigment epithelium (RPE) expressed SIRT4, SIRT6, and SIRT7. SIRT4 and SIRT7 were strongly positive in the macula and peripheral retina but not in the outer nuclear layer (ONL) [51].

In the mouse retina, SIRT6 is expressed in all retinal layers and its levels are higher in this tissue compared to brain, heart, liver, or kidney [52]. In humans, SIRT6 is expressed in macula, nonpigmented ciliary body epithelium, ciliary muscle, retinal pigment epithelium, optic nerve fiber, and neurosensory retina except the inner limiting membrane. SIRT6 expression was observed in retinoblastoma [48]. SIRT6 controls the levels of histone H3K9 and H3K56 acetylation. Its deficiency causes major chromatin changes in the retina that are accompanied by marked changes in expression of metabolic genes including GLUT1 and metabotropic glutamate receptor Grm6 and severe functional impairment in the SIRT6-KO retinas. These mice showed profoundly impaired electroretinogram (ERG) [52].

## 4. Role of Sirtuins in Glaucoma

Glaucoma and optic neuropathy are the leading chronic neurodegenerative disorders over the age of 40 and are associated with increased intraocular pressure, ischemia, oxidative stress, and deprivation of neurotrophic factors [53, 54]. Retinal ganglion cell (RGC) transmits light signals from the neural retina (where the light is captured and converted to electrical impulse) to the visual processing centers of the brain. Ischemic condition in glaucoma leads to hypoxia with increased apoptosis of RGC [55].

SIRT1 was first linked with hypoxia inducible factor (HIF) activity in hepatoma cells, where HIF-2 was acetylated at its C-terminal and consequential decrease in its transcriptional activity. SIRT1 activation reverses the acetylation of HIF-2 and increases its transcription and erythropoietin production [56]. In HEK293 cells, SIRT1 binds to HIF-1, deacetylates at Lys674 and blocks its association with the transcriptional coactivator, p300 [57]. Hypoxia affects NAD^+^/NADH ratio that in turn suppresses SIRT1 activity followed by acetylation and activation of HIF-1 [57]. SIRT1 binds to both HIF-1*α* and HIF-2*α*. In transfected HEK293 cells, HIF-2*α* competes with HIF-1*α* for SIRT1 binding. In support of this, erythropoietin-enhancer and vascular endothelial growth factor promoter reporter analysis showed that SIRT1 facilitated the transcriptional activity of HIF-2*α*, whereas it repressed HIF-1*α* activity. This study noted that SIRT1-mediated hypoxic responses appear to be dependent on the *α* subunit of HIF-1 [57]. Our earlier cell culture study showed that SIRT1 protects the hypoxic RGCs through inhibition of caspase-3 [54].

The Stress-activated Protein Kinase (SAPK)/-c-jun N-terminal kinases (JNK1/2/3) are important signaling kinases that are elevated in neurodegenerative diseases including glaucoma [58]. Differentiated hypoxic RGCs showed that blockade of SIRT1 has higher SAPK/JNK activity whereas inhibition of JNK (SP600125) showed higher SIRT1 activation in [17, 54]. This explains the SIRT1 role in balancing the proapoptotic versus antiapoptotic function. In addition, a study in a rat model of optic nerve axotomy found a direct correlation between SAPK/JNK and induction of apoptosis [59, 60].

In an optic nerve crush injury model, SIRT1 overexpressing (SIRT1-KI) mice had significantly higher RGC numbers compared with severe RGC loss in wild-type [61]. In a similar model, treatment of mice with 250 mg/kg of resveratrol (SIRT1 activator) attenuated the loss of RGC function by preserving pupillary light responses. However, SIRT1-KO mice did not show any effect after resveratrol treatment [61]. Another optic nerve injury study on calorie restricted rats showed decreased SIRT2 mRNA levels compared to the normal diet group but had no effect on survival of RGCs [62]. Retinal ganglion cells in mice pretreated with resveratrol showed protective effect with altered expression of SIRT1 but had minimal alteration in the expression of SIRT2 and SIRT5 followed by optic nerve crush [63].

Glaucomatous human retina showed 2-fold increased expression of SIRT3 compared to normal retina. In addition, human glaucomatous retina showed increased expression of SIRT1, SIRT3, SIRT6, and SIRT7 in the glial fibrillary acidic protein (GFAP) positive astroglia compared to age-matched nonglaucomatous controls [64].

## 5. Role of Sirtuins in Age-Related Macular Degeneration (AMD)

AMD, a common cause of blindness in the elderly population, is characterized by either the presence of drusen (dry AMD) or vascular epithelial growth factor- (VEGF-) induced choroidal endothelial cell proliferation with associated leakage (exudative or wet AMD) [65]. Oxidative stress and hypoxia induce several pathological changes in the retina including apoptotic cell death, dysfunction of the retinal pigment epithelial (RPE) cells, accumulation of lipofuscin, formation of drusen, and impairment of Bruch's membrane [66, 67]. SIRT1 deacetylates and activates HIF-2*α* and regulates VEGF-A promoter [61]. In our earlier study on hypoxic choroidal endothelial cells, we found that SIRT1 regulates vascular endothelial growth factor-A (VEGF-A) through the activation of HIF-2*α*. Increased VEGF levels in hypoxic cells and the subsequent decrease after the activation of SIRT1 establish a relation between SIRT1 and HIF-2*α* [68]. Similarly, resveratrol treatment inhibits hypoxic choroidal endothelial cell proliferation through SIRT1-dependent pathway at higher dosage [65].

Three octogenarian patients with AMD who fail to respond to anti-VEGF therapies treated with oral resveratrol showed dramatic short-term anti-VEGF type effect including anatomic restoration of retinal structure with an improvement in choroidal blood flow by near IR multispectral imaging. The improvement of visual function mirrors the effect seen anatomically with added benefit of RPE function and lasted for more than one year when taken daily [69]. In another recent study, Richer et al. reported broad bilateral improvements in ocular structure and function in three patients with AMD over a long-term follow-up of two to three years suggesting its efficacy in AMD [70]. Retinal photo toxicity which is another cofounding factor is associated with AMD. Oral administration of resveratrol showed protective effect against phototoxic degeneration of the mouse retina in vivo via activator protein-1 activation [71].

Cao et al. demonstrated that one of the constituents of drusen is amyloid beta (A*β*) (controlled by activation of SIRT1) induced inflammation in AMD. Amyloid beta induced changes in retinal pigment epithelial cell morphology while barrier integrity was balanced by SIRT1 activation by suppressing nuclear factor kappa-B (p65 subunit). The reduction in the nuclear factor kappa-B activation further decreased the inflammatory cytokine expression of interleukin-8 (IL-8), interleukin-6 (IL-6), and matrix metalloproteinase-9 (MMP-9) [72].

## 6. Role of Sirtuins in Optic Neuritis

Optic neuritis is an inflammatory optic neuropathy commonly associated with multiple sclerosis [73, 74]. It is a myelin sheath disease with lesions typically located in the optic nerve, brain and spinal cord, or cranial nerves. Normally, myelin helps electrical impulses travel quickly from the eye to the brain, where they are converted into visual information. Optic neuritis disrupts this process and affects vision [75]. Intravitreal injection of SIRT1 agonists inhibits RGC loss in a dose-dependent manner by inducing SIRT1 activity in mice with optic neuritis. This neuroprotective effect is blocked by sirtinol [46, 76]. Resveratrol represents a promising neuroprotective therapy for optic neuritis and traumatic optic neuropathies. Both SIRT1 overexpression and resveratrol treatment reduce the levels of superoxide accumulation in optic nerves following crush injury [61]. Since protective effect of resveratrol on endothelial and cancer cell is dose-dependent caution is necessary in using it as a therapeutic agent [65, 77]. In contrast to SIRT1 overexpression, in an established mouse model of multiple sclerosis, SIRT1 inactivation increased the production of new oligodendrocyte progenitor cells in the adult mouse brain; ameliorated remyelination; and delayed paralysis [78].

## 7. Role of Sirtuins in Neurodegenerative Diseases

Detailed description of sirtuins protective role in other neuronal diseases has been described in other literature. In a mixed culture of neurons and microglia, SIRT1 deacetylates (at p65 subunit) and reduces NF-kB signaling that protects neurons from amyloid beta induced toxicity in microglia [79]. Ischemic preconditioning and resveratrol treatment reduced neuronal injury of hippocampal CA1 after NMDA challenge in slices and global cerebral ischemic in rats [80, 81].

The inhibition of NF-kB by SIRT1 contributes neuroprotection similar to the effect of glucoside against Alzheimer's disease and ischemia [82, 83]. Resveratrol attenuates neuronal degeneration and death in animal models of Alzheimer's disease and Parkinson's disease associated with the cerebral accumulation of *β*-amyloid and *α*-synuclein, respectively [84, 85]. In humans, phase II and phase III clinical trials evaluate the usage of resveratrol in Alzheimer's disease patients. The primary outcome of these trials was evaluation of brain imaging, cerebrospinal fluid marker analysis, and cognitive report [86, 87]. SIRT2 inhibitors are capable of postponing the axon degeneration in Parkinson's disease models due to their presence in cell bodies of neurites and growth cones in axons [62]. Loss of sirtuin 4 (SIRT4) in mice leads to decreased glutamate transporter expression and function in the brain, which can cause increased excitotoxic effects. Loss of glutamate transport function is implicated in epilepsy, traumatic brain injury, and amyotrophic lateral sclerosis [88].

Another common neurodegenerative disease is Parkinson's disease (PD) which affects the elderly population in industrialized countries and is characterized by tremor, postural instability, and rigidity. SIRT1 overexpression protects against Parkinson's disease. SIRT1 activates heat shock factor (HSF1) that impacts the transcription of molecular chaperones including heat shock protein 70 and homeostasis of other cellular proteins [18, 89]. In contrary to this, SIRT2 inhibitors showed dose-dependent effect of *α*-synuclein mediated toxicity in cell culture models. This effect was mediated by increasing the size of aggregates but reduced the number of synuclein aggregates. SIRT2 showed protective effect in a mouse model of PD using 1-methyl-4-phenyl-1,2,3,6-tetrahydropyridine (MPTP). Activated SIRT2 in MPTP-induced stress cause Foxo3a deacetylation which lead to increased levels of the proapoptotic factor Bim and trigger neuronal death [90]. In a similar model, absence of SIRT5 increased dopaminergic degeneration whereas presence of SIRT5 maintains the function [91].

Huntington disease occurs due to expansion of CAG repeats codes for glutamine residues which affects conformation and aggregation of huntingtin protein [92]. SIRT1 was shown to be protective against this disease in cell culture models and drosophila systems. In a chemically induced mouse model of HD using 3-nitropropionic acid, treating the animals fed with resveratrol decreased cognitive and motor defects. However, in the N171-82Q transgenic mouse model with overexpressed truncated huntingtin protein, resveratrol treatment did not improve the survival [93, 94]. Similar to SIRT1, SIRT2 inhibition was shown to be protective against HD in cell culture and mouse models. However the mechanism is linked with a decrease in the sterol biosynthesis pathway [95]. Recently, viniferin, another resveratrol derivative, was found to be protective by increasing SIRT3 levels that activate/deacetylate manganese superoxide dismutase (MnSOD) and liver kinase B (LKB) [96].

## 8. Role of Sirtuins in Metabolic and Health Span

Sirtuins promotes cellular longevity using calorie restriction (CR). It is associated with reduced food consumption of an organism compared to normal consumption. This increases nicotinamide adenine dinucleotide (NAD^+^) levels in liver which in turn activates SIRT1 [97]. SIRT1 also activates PGC1*α* which results in mitochondriogenesis [97]. A decline in mitochondrial activity upon aging is a causative factor in many age-related diseases [98]. SIRT2 has been found to regulate metabolism by deacetylating and stabilizing phosphoenolpyruvate carboxykinase (PEPCK1), which is the rate limiting enzyme for gluconeogenesis, linking SIRT2 with type II diabetes [99]. In response to nutrient deprivation and energy expenditure it promotes lipolysis and inhibits adipocyte differentiation through deacetylation of FoxO [99, 100]. SIRT2 deacetylates FoxO3 and increases its transcription after calorie restriction in mice and after hydrogen peroxide treatment in kidney cells thus reducing cellular levels of ROS [101].

Recently, it has been reported that SIRT3 has been found to regulate many aspects of mitochondrial function, such as metabolism, Adenosine triphosphate (ATP) generation, and modulation of the response to oxidative stress using acetyl-coA synthetase 2 (AceCS2) and glutamate dehydrogenase (GDH). A shift from dependence on liver glycolysis, facilitated by GDH and AceCS2 activity, has been implicated in calorie restriction (CR), suggesting a role of SIRT3 in reprogramming metabolism during CR to allow respiration [102–104].

However, presence of SIRT4 inhibits GDH in pancreatic beta cells and opposes the effects of CR. SIRT4 deficient or CR mice are insensitive to phosphodiesterase (an enzyme that cleaves ADP-ribose and is essential for ADP-ribosylation). This indicates that, in beta cell mitochondria, SIRT4 repress the activity of GDH by ADP-ribosylation, thereby downregulating insulin secretion in response to amino acids, effects that are alleviated during CR [105]. SIRT5 and SIRT6 are sensitive to CR and are induced by low calorie stress. Under CR, upregulated SIRT5 in liver leads to increased physiological needs for nitrogen disposal due to increased amino acid metabolism [106].

SIRT6 is involved in human telomere and genome stabilization, gene expression and DNA repair, glucose homeostasis, and inflammation [107–109]. Aging phenotype was observed in SIRT6 knockout mice where they showed increased transcription of NF-kB that trigger increased apoptotic resistance and cell senescence. This is reversed by the inhibition of RelA subunit of NF-kB. This finding provides the evidence that SIRT6 binds NF-kB subunit of RelA and modulates NF-kB target genes [110]. SIRT6 deacetylate H3K9 and control expression of multiple glycolytic genes lactate dehydrogenase (LDH), triose phosphate isomerase (TPI), aldolase and phosphofructokinase (PFK1). SIRT6 deficient cells showed elevated HIF-1*α* activity and elevated glucose uptake with an increased glycolysis and reduced mitochondrial respiration. Based on these functions, SIRT6 can serve as a biomarker for metabolic diseases [107].

## 9. Role of Sirtuins in Cardiovascular Diseases

SIRT1 deficient mice showed developmental defects in the heart and are embryonically lethal. However, heterozygous SIRT1 deficient mice showed absence of fibrosis and decreased cardiomyocyte size [111]. At cellular level, SIRT1 promotes vascular relaxation by activating endothelial nitric oxide synthase (eNOS). SIRT1 interacts with NF-kB by inhibiting its signaling and proinflammatory cytokine release in endothelial cells [109, 112, 113]. SIRT1 promotes angiogenesis by increasing VEGF expression through hypoxia signaling. Pharmacological inhibition of SIRT1 using sirtinol decreases VEGF levels [68]. Inhibition of SIRT1 increases thrombosis by reducing tissue factor activation through the pathway of Peroxisome Proliferated-Activated Receptor *δ*, cyclooxygenase-2 derived prostacyclin, or NF-kB [114, 115].

Animal studies found that resveratrol (2.5 mg/kg) prevents the cardiac dysfunction in spontaneously hypertensive rats [116]. In a minipig model of heart failure, transplanted stem cell cultures from wild type showed more proliferation compared to transplants from SIRT1 knockout stem cell sheets. They also showed less cytokine release compared to wild-type cells. Similar to this, the Lewis rat model of heart failure also showed lesser cardiac function in a SIRT1 knockout stem cell transplants. These experiments explain that SIRT1 mediates regenerative capability of stem cells in heart failure [117].

SIRT3 knockout mice also showed the signs of cardiac hypertrophy. SIRT3 regulates mitochondrial function and the inhibition of SIRT3 causes mitochondrial dysfunction, which in turn causes reduced oxygen consumption. Overexpression of SIRT3 reduces ROS levels and mitochondrial DNA damage underlying with vascular inflammation in atherogenesis [109]. SIRT3 deacetylates/activates superoxide dismutase 2 (SOD2) which further increases the deacetylation of transcription factor forkhead box o3a (FOXO 3a) and protects against cardiac hypertrophy [118]. In a recent study, decreased SIRT3 in human pulmonary artery smooth cell was associated with an induction of transcription factors HIF-1alpha, signal transducer and activator of transcription-3 (STAT3), and nuclear factor of activated T-cells cytoplasmic 2 (NFATc2) and was associated with pulmonary arterial hypertension [119].

Similar to SIRT3, SIRT6 knockdown mice showed cardiac hypertrophy and the overexpression of SIRT6 rescues cardiac hypertrophy. SIRT6 interacts with stress reactive kinase c-jun and suppresses the promoter of genes in IGF-signaling pathway. This inhibition further decreases the expression of genes in IGF-akt signaling that participates in the progression of heart failure [120, 121]. Interaction of SIRT6 with a transcription factor NF-kB at Rel A subunit protects against inflammation [110].

In a recent study by Araki et al. [122], myocardial infarction and hindlimb ischemic mouse models showed high levels of SIRT7 expression, whereas SIRT7 knockout mice were more susceptible to cardiac rupture after myocardial infarction and delayed blood flow recovery after hindlimb ischemia. In vitro mechanistic evaluation provided the evidence that cardiac fibroblasts derived from these SIRT7 knockout mice showed reduced transforming growth factor beta (TGF-*β*) signaling and TGF beta receptor I protein compared to wild-type mice derived cells. They showed low levels of fibrosis related genes [122].

## 10. Role of Sirtuins in Cancer

SIRT1 role in cancer was widely studied and described in detail in earlier literature. We will briefly describe it in our review. It plays a dual role in promoting angiogenesis and acts as a tumor suppressor. SIRT1 is involved in genome stability, inflammation, DNA repair, and apoptosis processes [123]. SIRT1 activates HIF-2*α* and Rel A/p65 subunit of NF-kB and promotes angiogenesis through the production of VEGF in choroidal endothelial cells [68]. In hepatocellular carcinoma, SIRT1 promotes accumulation of HIF-*α* and activates transcription of HIF-1alpha target genes [124]. A recent study found mutations in the SIRT1 gene in several breast cancer cell lines that were related to breast cancer progression [125]. SIRT2 plays a dual role like SIRT1. SIRT2 knockout mice developed tumors after 10 months of age compared to wild-type mice [126]. They developed mammary tumor and hepatocellular carcinoma [127]. In addition to tumor suppression, knockdown of SIRT2 or pharmacological inhibition provides an antiproliferative effect in cancers. A recent study on neuroblastoma cell line found that SIRT2 inhibition downregulates C-MYC and N-MYC oncogenic proteins [128]. Tenovin-D3 another SIRT2 inhibitor was found to increase the tumor suppressor protein p21 [129]. SIRT2 activates lactate dehydrogenase A (LDH-A) and the increased amount of LDH-A was noted in many cancer cells. Thus inhibition of SIRT2 disrupts the cancer metabolism [123, 130].

SIRT3 was reported to be protective in several cancers like oral carcinoma, breast ovarian, and renal cancers [131]. SIRT3 was found to decrease ROS and ROS participates in HIF and akt signaling that plays a critical role in cell proliferation [118]. In vitro studies in human cancer cells revealed that overexpression of SIRT3 decreases the cell proliferation [132]. He et al. [133] found that SIRT3 levels were correlated with clinical features such as metastasis and tumor size in breast cancer. In contrast, recent meta-analysis on 14 studies with 2165 cancer patients assessed the relation between SIRT3 immunohistochemical expression and their respective survival and clinical pathological characters. This study did not find any correlation between SIRT3 expression and clinical pathology. They also concluded that SIRT3 is associated with prognosis and clinical parameters in specific cancers [134].

The inhibitory role of SIRT4 on GDH makes SIRT4 as a tumor suppressor. SIRT4 was found to be downregulated in many cancers. SIRT4 knockout mice developed lung tumors within 18–26 months compared to wild-type SIRT4 mice [135]. SIRT6 also serve as a tumor suppressor by regulating HIFs [107] and NF-kB. SIRT6 plays a role in genomic instability and DNA repair and inflammation explains SIRT6 participation in cancer. A study explains that immortalized mouse embryonic fibroblasts (MEF) cells from SIRT6^−/−^ mice developed more tumorigenicity than MEF from SIRT6^+/+^ mice. They suggest that this is mainly due to reprogramming of metabolism through two transcription factors HIF-1alpha and MYC [136].

So far, various microRNAs (miRNA) have been reported to bind SIRT1 and modulate SIRT1 deacetylation target genes. However, recent in vitro and in vivo analysis found that SIRT7 promotes gastric cancer growth by deacetylating H3K18ac at the promoter of miRNA-34a, whereas reducing/knocking down SIRT7 inhibits the cancer cell growth. They also showed G2/M accumulation in the cell cycle [137]. In addition ovarian and breast cancer cells showed high levels of SIRT7 and reducing SIRT7 downregulated cancerous cell growth and impacts apoptotic related proteins (NF-kB) [138, 139].

## 11. Conclusion and Future Aspect

Although limited, it is evident from experimental studies that sirtuins promote survival of RGCs, confer protection against cell death, and are important players in eye-related neurodegenerative diseases discussed above. Current data therefore supports the concept that modulation of sirtuin activity to provide neuroprotection in these diseases may have therapeutic implications. Yet, drug delivery still remains a major challenge in ocular treatment, especially for diseases affecting posterior and anterior segment of the eye. Systemic administrations fail to achieve a therapeutic concentration of drug due to the presence of blood-aqueous and blood-retinal barriers. In contrast, intravitreal administration can achieve higher concentration of drug to treat posterior segment diseases, but the process is very painful and suffers from poor patient compliance. Despite these challenges, continued efforts in this direction have helped to find numerous strategies to improve drug delivery system [140]. These strategies include formulating drugs into implants and use of micro- or nanoparticulate and hydrogel-based systems. Transporter targeted prodrug approach has also been described to deliver drugs to both the anterior and posterior segments of the eye. Noninvasive drug delivery methods utilizing ultrasound, iontophoresis, and microneedle based devices have been promising [140]. In addition, recently, the delivery system of a sirtuin-activating agent, resveratrol, was developed and patented by Allergan Inc. The inventors have shown prolonged retinal ganglion cell survival and neuroprotection by administration of resveratrol embedded in biodegradable polymer like poly-lactic-co-glycolic acid (PLGA) and intend to use this formulation for the treatment of posterior segment disorders like AMD and macular edema.

Future studies are needed to better understand and elucidate the molecular role of sirtuins and identify their substrate partners/cofactors and the intracellular pathways that regulate their activity in different disease models. Nonetheless, it is essential and plausible to develop and test (clinical trials) specific pharmacological activators or inhibitors of sirtuins that may mediate neuroprotection and serve as beneficial strategy for treatment of the neurodegenerative diseases of the eye [141, 142].

## Figures and Tables

**Table 1:** Certain major functions of sirtuins.

Name of the sirtuin (SIRT)	Functions	Subcellular localization
SIRT1	Cellular longevity, tumor promoter, tumor suppressor, inflammation, oxidative stress, glucose homeostasis, cell adhesion, cell metabolism	Nucleus
SIRT2	Mitotic check point, tumor promoter, tumor suppressor	Cytoplasm/nucleus
SIRT3	Tumor suppressor, tumor promoter, mitochondrial oxidation, stress responsive deacetylase	Mitochondria
SIRT4	Glutamine catabolism, TCA cycle, ADP-ribosylation	Mitochondria
SIRT5	Glycolysis, cancer metabolism, fatty acid oxidation, lysine succinylation, malonylation, glutarylation	Mitochondria
SIRT6	Telomere and genome stability, DNA repair, inflammation, glucose homeostasis	Nucleus
SIRT7	Ribosomal production	Nucleus (nucleoli)
